# Sheffield Shield Cricketers Live Longer than the Age-Matched General Australian Male Population

**DOI:** 10.1007/s43465-023-00925-3

**Published:** 2023-09-21

**Authors:** Nathan Luies, Jessica J. Orchard, Tim Driscoll, Saaz Kaur Sahdra, Jonathan Cheng, Angus J. Davis, John W. Orchard

**Affiliations:** 1 Perth Orthopaedic and Sports Medicine Centre, 31 Outram St, West Perth, WA 6005 Australia; 2 Sydney School of Public Health, Faculty of Medicine and Health, The University of Sydney, Edward Ford Building, Fisher Road, Sydney, NSW 2006 Australia; 3 School of Computer Science, Faculty of Engineering, The University of Sydney, Building J12, I Cleveland Street, Darlington, NSW 2008 Australia; 4 South Western Sydney Clinical School, University of New South Wales, Burnside Drive, Warwick Farm, NSW 2170 Australia

**Keywords:** Life expectancy, Sport, Athletes, Australia, Cricket, Mortality

## Abstract

**Background/objectives:**

Previous studies have shown a trend that elite athletes tend to live longer than the general population, which has been attributed to the “healthy worker hire effect” and the health benefits of exercise. There have not been any previous studies looking at survival of elite cricketers with the general population as a reference cohort. This study aimed to compare the annual mortality rates of current and retired elite male Australian cricket players to that of the age-matched general Australian male population.

**Methods:**

Analysis of publicly accessible dates of birth, death, and cricket debut data for male Australian Sheffield Shield cricket players who played before 2022 and had not died before 1971. Included persons were Sheffield Shield players who lived primarily in Australia during and after their cricket careers. Death rates from 1971 to 2021 (inclusive) were compared to the general Australian male population.

**Results:**

1824 Sheffield Shield players had not died prior to 1971 (798 had played before the 1971 season, 1026 debuting subsequently). There were 586 deaths in the 51 years of observations, compared to 825 expected deaths, giving a Standardized Mortality Ratio of 0.71 (95% CI 0.63–0.80).

**Conclusion:**

Elite Australian male Sheffield Shield cricket players outlive the general male population with lower death rates. This is probably due to a combination of the healthy worker hire effect and the health benefits of exercise. This study provides evidence that in terms of longevity, it is safe to play elite-level cricket in Australia.

**Graphical Abstract:**

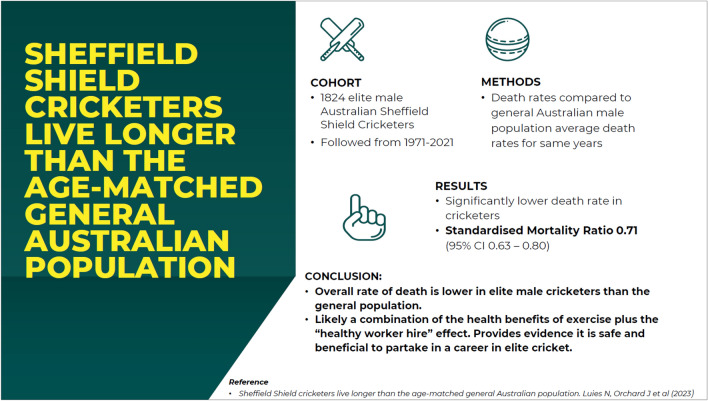

## Introduction

Olympians and other elite athletes globally exhibit a longer life span compared to the general population [1–3]. This is likely to be at least partly because persons able enough to participate competitively in sports at the elite level are less prone to premature death than members of the general population, essentially an example of the “healthy worker hire effect” [4, 5]. It might also be the case that participation at the highest level of athletic competition has a positive impact on one’s longevity, for example due to the known survival benefits of exercise [6]. In either situation, this longevity advantage may be more specifically due to lower incidence of cancer and cardiovascular disease [7], which are the leading causes of death in the general population. Few studies of longevity involve Australian athletes, although a recent study on elite Australian Rules football players demonstrated death rates to be lower than that of the general population [8].

There is a paucity of studies looking specifically at the longevity of cricketers. A series of studies compared longevity of left- and right-handed cricketers which demonstrated an increased likelihood of death from unnatural causes, such as war, for left handers. However, there was no significant association between overall mortality and handedness, and no non-cricket controls were included in these studies [9, 10]. It has also been hypothesized that male Test cricketers are more prone to suicide than the general population, but a subsequent study did not support this [11, 12]. As cricket is an outdoor sport with higher UV exposure, cricketers may also be more susceptible to skin cancer, which may impact mortality. However, very little data on skin cancer in cricketers has been published [13], and this is a potential area to explore in future research.

A recent spate of high-profile deaths in retired male cricketers in 2022 has raised the question of whether cricketers have normal life expectancy. Longevity data for elite Australian male cricket players have not previously been reported. We aimed to investigate this by studying Sheffield Shield cricketers, for whom there is comprehensive publicly-available lifespan data. Similar data were not available for female cricketers and so the focus of this study was males only.

Our hypothesis was that male players participating in elite Australian cricket would demonstrate a lower incidence of mortality relative to the general population. We aimed to determine the annual age-matched death rates of current and retired elite male Australian cricket players compared to the general population in the years 1971–2021 inclusive, years for which age-specific death rates for the general population are publicly available [8].

## Materials and Methods

The research solely utilized publicly-accessible data. Birth and death information of players, along with year of debut, was accessed through free online sources such as Wikipedia [14], Espncricinfo [15], and Cricketarchive [16]. Age-adjusted mortality rates for the Australian population were accessed from the Australian Bureau of Statistics [17].

The inclusion criteria were: Sheffield Shield players who played at least one game prior to the 2022–2023 season, lived primarily in Australia during and after their cricket careers, and had not died prior to 1971. The study was restricted to male players only as there were not enough comparative data for female players.

The Sheffield Shield is an elite Australian domestic 4-day cricket competition. The inaugural edition was contested between Victoria, New South Wales, and South Australia during the 1892–93 season. Subsequently, the remaining Australian states joined in a graduated manner: Queensland in 1926–27, Western Australia in 1947–48, and Tasmania in 1977–78. While the competition was disrupted during the First and Second World War years, first-class games were still held. The study was restricted to Sheffield Shield players, as this competition had very complete statistical records and clarity of which games were included, whereas there is some ambiguity about first-class status of matches outside the Shield competition.

Espncricinfo pages [15] were used in early 2023 to determine which players had made Shield debuts and the year of debut. The first year of the season was considered the year of the debut, because the season of debut was listed rather than a date. For example, if a player made their debut in the 1971–1972 season, they were considered to have made their debut in 1971. Wikipedia [14] was used as the source of the date of birth, date of birth and current living status as of January 2023 for all players who made their debut before 2022 and had not died before the start of 1971.

There was a small subgroup of Sheffield Shield cricketers who were born overseas. The study included those who were dual citizens or spent the majority of their life in Australia despite being born overseas. Those who were short-term residents of less than 5 years were excluded.

Players joined the cohort of analysis upon debut, with follow-up continued for each player until the end of 2021 or death. Actual death rates of the cohort were compared to expected deaths by referencing the general population age-specific death rates, arranged in 5-year groupings. Both per year and per decade actual death rates were compared.

The control group was the Australian male population in each year of the study (1971–2021) with data freely sourced from the Australian Bureau of Statistics online Stat Data Explorer. The dataset used was “Deaths, Year of Registration, Age at Death, Age-specific death rates, Sex, and Australia” using filters: All Years (1971–2021), Sex = Male, and Measure = Age-specific death rate [17]. Expected deaths per year were calculated by multiplying the number alive in the cohort in each 5-year age group (ages 15–19, 20–24, 25–29….0.95–99, 100 and over) by the average death rate for that age group based on the population data.

Taylor series expansions [18] in Excel (Microsoft, Seattle) were used to calculate confidence intervals for the standardized mortality ratios (SMRs).

Since the analysis involved publicly-available data without any individual identification, the study was deemed exempt from ethics considerations. We utilized our Institutional Online tool to verify that the study would be classified as “negligible risk,” meaning that it did not necessitate formal ethics committee evaluation with confirmation that other published studies only using publicly available data had not required ethics committee assessment [19–22].

## Results

There were 3417 players considered for inclusion in the analysis, listed on Wikipedia [14] as having played for one of the six Australian states. Of these players, 54 were excluded because they were overseas players (not Australians) and 1337 were excluded due to dying prior to 1971. There were 210 first-class players and 89 other players excluded because they did not ever play a Sheffield Shield match in the official competition. There were also three Australian Sheffield Shield players who were excluded because they were presumed dead in 2022 (but without any known date of death), and without the certainty that they were alive in 1971.

The final eligible cohort comprised 1824 Australian males who played Sheffield Shield cricket and who had not died prior to 1971. Of this final cohort, 798 had played before the 1971–72 season, with the other 1026 debuting between the 1971–72 and 2021–22 seasons. Cohort numbers for each year are shown in Table 1, increasing each year by the number of additional debut players, minus the number of players who had died in the preceding year.

**Table 1:** Cohort exposure by year

	Y0	Y1	Y2	Y3	Y4	Y5	Y6	Y7	Y8	Y9
1970s		798	805	809	815	818	826	855	877	886
1980s	885	896	924	929	943	945	956	962	966	968
1990s	982	1011	1016	1032	1043	1050	1052	1058	1068	1079
2000s	1078	1094	1098	1108	1118	1121	1123	1131	1141	1153
2010s	1162	1187	1193	1206	1207	1216	1218	1226	1237	1246
2020s	1242	1244								

There were 586 deaths in the 51 years of observation. There were only 4 years where expected deaths (Table 2) were lower than actual deaths (Table 3), with the other 47 years having fewer deaths than expected. When considered as isolated years, there were only 2 years where the reduced number was statistically significant. However, when considered by decade (Table 4), every decade had a significantly-reduced death rate at the 95% confidence interval level.

**Table 2:** Expected deaths by year

	Y0	Y1	Y2	Y3	Y4	Y5	Y6	Y7	Y8	Y9
1970s		16.4	16.2	16.3	17.4	15.1	16.1	16.0	15.9	16.2
1980s	16.4	16.6	17.6	16.2	14.6	15.4	14.2	15.0	15.0	15.5
1990s	15.2	15.4	16.3	16.3	16.6	15.9	15.3	15.5	15.2	15.0
2000s	14.7	14.9	15.4	15.3	15.9	15.2	15.9	16.0	16.2	15.8
2010s	15.2	15.7	16.2	16.3	16.9	17.3	16.9	17.6	18.4	20.0
2020s	19.2	20.8

**Table 3:** Actual deaths by year

	Y0	Y1	Y2	Y3	Y4	Y5	Y6	Y7	Y8	Y9
1970s		13	9	10	16	8	7	9	9	15
1980s	14	7	17	18	15	15	8	14	11	9
1990s	8	9	12	15	12	16	10	9	13	13
2000s	6	16	11	8	14	12	10	11	11	12
2010s	7	11	12	12	11	15	7	7	14	11
2020s	14	13

**Table 4:** Standardized Mortality Ratio (by decade) compared to the general male Australian population

Year	Expected deaths	Actual deaths	Standardized mortality ratio (SMR)	95% CIs	
1971–1980	161.9	110	0.68	0.53	0.87
1981–1990	155.4	122	0.79	0.61	1.00
1991–2000	156.3	115	0.74	0.57	0.94
2001–2010	155.8	112	0.72	0.56	0.92
2011–2020	174.6	114	0.65	0.51	0.83
2021	20.8	13			
Total	825	586	0.71	0.63	0.80

### Deaths Considered by Age

**Fig. 1:**
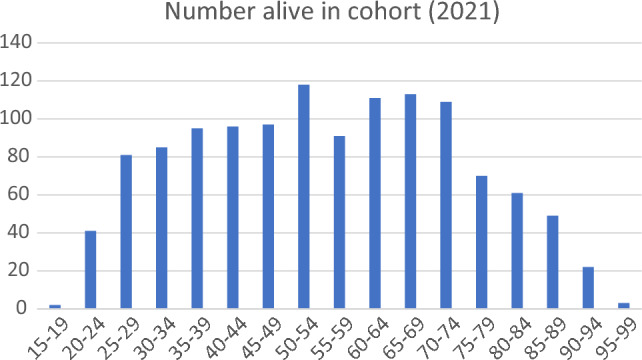
Age range of the 2021 cohort (living players and ex-players)

**Fig. 2:**
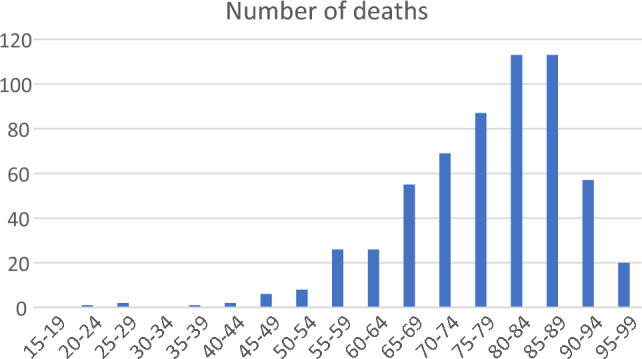
Age range (at death) of players who died, 1971–2021 inclusive

The age spread of players and ex-players alive in the cohort in 2021 is shown in Fig. 1. Deaths at each age group for those who died in the 51-year period are shown in Fig. 2.

Deaths in younger cricketers were very uncommon. Only three players died in their 20s, with two of these being cricket related (one was a neurological injury in a match, one a cardiovascular condition at training) [14]. A player in his 20s died at home from a neurological condition. Only one player died in his 30s, as a result of a motor vehicle accident [14]. 20 ex-players died between the ages of 95 and 99 (Fig. 2). The most common age decade for dying (Fig. 2) was players in their 80s.

## Discussion

Sheffield Shield players who were alive in the 51-year period from 1971 to 2021 outlived the age-matched general Australian male population. Although cause of death was not able to be reported or analysed in this study, we can confirm that the overall rate of death is lower in elite male cricketers than the general population. This appears likely to be due either to the health benefits of exercise or the “healthy worker hire effect”, most likely a combination of both [5]. These findings are keeping with previous research where Olympians and other elite sporting cohorts have longer life spans than the general population [4].

We believe that using expected deaths per year in comparison to the general population was a more precise indicator of longevity than life expectancy or age at death. A player must have been alive when he debuted in Sheffield Shield cricket, so we chose to only include players in the study from the year of debut. The interpretation of the findings is also influenced by the “healthy worker hire effect” in that active players in their 20s and 30s who are very fit and healthy are compared to the general population that includes both disabled and unwell individuals. This is not a limitation in terms of the study hypothesis, but is a limitation if the intention is to examine whether males will have a lower incidence of mortality if they play Sheffield Shield cricket compared to if they did not play Sheffield Shield cricket. In that instance, the healthy worker hire effect would be relevant (essentially this is an issue of incompletely controlled confounding). This is a problem for almost every study in this genre and probably accounts for some of the difference between mortality rates of elite athletes and the general population. The extent of any “healthy worker hire effect” is difficult to measure. The study was not subject to the “healthy worker survivor effect” bias [23], whereby those who remain employed tend to be healthier than those who leave employment, as the study captured every single player who played Sheffield Shield cricket during the years of analysis.

The reduced death rates compared to the general population in this study (SMR 0.71) are similar to those of a recent cohort of elite Australian Rules footballers (SMR 0.79) [8]. There have also been studies showing some athletes (powerlifters) [24] and celebrity groups (musicians) failing to outlive the general population [25].

As a follow-on study from a similar analysis of mortality of male Australian Rules Footballers compared with the general population [8], this study also utilized publicly available data and relied on the seemingly comprehensive death data available on Wikipedia. The study’s methodology could be replicated by any research group interested in conducting a similar analysis, and the data could be updated in the future using similar techniques. Reviewing player Wikipedia pages alongside espncricinfo and Cricketarchive data of Sheffield Shield players suggests that the data for individuals is reliable. We excluded only three cases where the year of death was likely to be missing (i.e. player is presumed dead with no known date). Although the creation and editing of Wikipedia pages, as well as the transfer of death data into a database, may result in data errors, we believe the risk is low. Therefore, we consider the number of deaths likely to be “missing” on Wikipedia and the related cricket references (Espncricinfo and Cricketarchive) to be very small.

Due to the lack of comprehensive data on the cause of death, we cannot provide insights into the rates of specific causes of death (such as suicide, skin cancer, heart disease and neurodegenerative disease). Therefore, we are unable to make any comments or draw conclusions on these topics. However, it would seem likely that the rates of cardiovascular disease and cancer (the two most common causes of death in Australia) would be lower than in the general population, perhaps due to the benefits of exercise and physical activity [7, 26].

In 2022, two renowned former Test cricket players passed away, one due to a fatal car accident while in his 40s, and the other from a cardiac arrest while in his early 50s. As of January 2023, only 14 deaths had been reported among this cohort in 2022, a similar number to the deaths in the years immediately preceding that one. It is possible that the expected deaths in 2022 will be higher than those of 2021, as the number of excess deaths in Australia had risen due to a high local rate of coronavirus deaths [27]. Complete analysis of the relative 2022 death rates can be undertaken when the final 2022 age-specific death rates are published by the Australian Bureau of Statistics [17].

## Conclusion

Overall, the overall death rate is lower in elite male Australian cricketers than in the general population and is in keeping with previous studies that demonstrate that elite athletes live longer. This is probably due to a combination of the health benefits of exercise and the healthy worker hire effect. This study provides evidence that in terms of longevity, it is safe to play elite-level cricket in Australia.

## Data Availability

This study used publically-available data. Statistical calculations and records of downloaded data from January 2023 would be available to researchers on request from the authors.
